# Biodegradation, Biosorption of Phenanthrene and Its Trans-Membrane Transport by *Massilia* sp. WF1 and *Phanerochaete chrysosporium*

**DOI:** 10.3389/fmicb.2016.00038

**Published:** 2016-01-29

**Authors:** Haiping Gu, Jun Lou, Haizhen Wang, Yu Yang, Laosheng Wu, Jianjun Wu, Jianming Xu

**Affiliations:** ^1^Institute of Soil and Water Resources and Environmental Science, Zhejiang Provincial Key Laboratory of Subtropical Soil and Plant Nutrition, Zhejiang UniversityHangzhou, China; ^2^Department of Civil and Environmental Engineering, University of Nevada, RenoNV, USA; ^3^Department of Environmental Sciences, University of California at Riverside, RiversideCA, USA

**Keywords:** phenanthrene, *Massilia* sp. WF1, *Phanerochaete chrysosporium*, biodegradation, biosorption, transport

## Abstract

Reducing phenanthrene (PHE) in the environment is critical to ecosystem and human health. Biodegradation, biosorption, and the trans-membrane transport mechanism of PHE by a novel strain, *Massilia* sp. WF1, and an extensively researched model fungus, *Phanerochaete chrysosporium* were investigated in aqueous solutions. Results showed that the PHE residual concentration decreased with incubation time and the data fitted well to a first-order kinetic equation, and the *t*_1/2_ of PHE degradation by WF1, spores, and mycelial pellets of *P*. *chrysosporium* were about 2 h, 87 days, and 87 days, respectively. The biosorbed PHE was higher in *P*. *Chrysosporium* than that in WF1, and it increased after microorganisms were inactivated and inhibited, especially in mycelial pellets. The detected intracellular auto-fluorescence of PHE by two-photon excitation microscopy also proved that PHE indeed entered into the cells. Based on regression, the intracellular (*K*_din_) and extracellular (*K*_dout_) dissipation rate constants of PHE by WF1 were higher than those by spores and mycelial pellets. In addition, the transport rate constant of PHE from outside solution into cells (*K_in_S/V_out_*) for WF1 were higher than the efflux rate constant of PHE from cells to outside solution (*K_out_S/V_in_*), while the opposite phenomena were observed for spores and mycelial pellets. The amount of PHE that transported from outside solution into cells was attributed to the rapid degradation and active PHE efflux in the cells of WF1 and *P*. *Chrysosporium*, respectively. Besides, the results under the inhibition treatments of 4°C, and the presence of sodium azide, colchicine, and cytochalasin B demonstrated that a passive trans-membrane transport mechanism was involved in PHE entering into the cells of WF1 and *P*. *Chrysosporium*.

## Introduction

Polycyclic aromatic hydrocarbons (PAHs) are typical persistent organic compounds (POPs) that mostly generated from the incomplete combustion of fossil fuels, waste incineration, forest and prairie fires, and industrial processes (Verdin et al., 2004; Bamforth and Singleton, 2005; Zeng et al., 2010). PAHs are widespread distributed in environments, such as air, soil, water, sediment, etc. (Kim et al., 2013; Cheng et al., 2014; Balasubramaniyam et al., 2015; Johnson et al., 2015). Because of their potential bioaccumulation and high toxicity (mutagenic, carcinogenic, teratogenic) to living organisms including human, animals, plants, and microbes (Kim et al., 2013; Cheng et al., 2014; Balasubramaniyam et al., 2015; Johnson et al., 2015), 16 PAH compounds, including phenanthrene (PHE), have been listed as priority pollutants by both the United States Environmental Protection Agency and European Union (Verdin et al., 2004). Consequently, great attention has been paid to study the behavior of PAHs in environments and to develop effective practices to remediate PAHs contaminated sites in the past decades.

Although PAHs are subject to a range of dissipation processes including volatilization, photooxidation, chemical oxidation, sorption, leaching, and biological process (Stringfellow and Alvarez-Cohen, 1999; Chen and Ding, 2012; Thion et al., 2012; Xu et al., 2013), biodegradation by microorganisms has been generally considered to be one of the primary means for the removal of PAHs from environment (Bamforth and Singleton, 2005; Haritash and Kaushik, 2009; Wang et al., 2009; Chen et al., 2010; Zeng et al., 2010; Xu et al., 2013). It was observed that for most microorganisms, the PAHs biodegradation process occurred intracellularly (Xu et al., 2013) and the trans-membrane transport of PAHs was the first step in biodegradation (Fayeulle et al., 2014; Li et al., 2014). In addition, the biosorbed PAHs in microorganisms might be readily accessible for biodegradation by stimulating the intracellular physico-chemical and biochemical processes with prolonging the incubation time (Chen et al., 2010; Ning et al., 2010; Chen and Ding, 2012; Thion et al., 2012). Since PAHs were toxic compounds to many microorganisms, the active efflux from cells to outside may occur as the most efficient detoxification way (Huang et al., 2014). Nevertheless, the mechanisms of PAHs entering into microorganisms and the fate of PAHs in cells have received considerably less attention (Stringfellow and Alvarez-Cohen, 1999; Verdin et al., 2005; Xu et al., 2013; Fayeulle et al., 2014). Hence, explorations of the trans-membrane transport mechanism and biosorption of PAHs, combined with biodegradation of PAHs are of great value in investigating the bioremediation of PAHs.

In the previous study, we isolated a PHE-degrading strain WF1 from an aged PAHs-contaminated soil in Jiangsu Province, China (31.604^o^N, 120.476^o^E), and it was identified as *Massilia* sp. based on the 16S rDNA analysis (GenBank accession number KF573748). It is noteworthy that the *Massilia* was one of the dominant species in the bacterial communities of PAHs or other organic pollutants (OPs) contaminated environments (Bodour et al., 2003; Ni Chadhain et al., 2006; Macías-Flores et al., 2009; Zhang et al., 2010; Gong et al., 2012; Silva et al., 2013). The researchers who reported the capacity of microbial consortium on OPs degradation suggested that *Massilia* might be involved in OPs degradation in the environment. But among them only Bodour et al. (2003) and Macías-Flores et al. (2009) obtained the strain *Massilia* sp. from the community; but still, they did not individually evaluate the OPs biodegradation with the isolated stains.

Since no detailed information about the role of *Massilia* sp. in the bioremediation of PAHs and other OPs is available, this research attempted to characterize the biodegradation dynamics, biosorption (active and inactivated microorganisms), and the mechanisms involved in trans-membrane transport of PHE by *Massilia* sp. WF1 in liquid cultures with PHE as the sole carbon source. Meanwhile, an intensively researched and effective model fungus for the degradation of PAHs and other OPs, *Phanerochaete chrysosporium* (Zhang et al., 2008; Chen and Ding, 2012), was also used in this research to compare the efficacy of PHE biodegradation, biosorption, and the trans-membrane transport mechanism between the fungi and the strain WF1.

## Materials and Methods

### Chemicals and Media

Phenanthrene (PHE; >99% purity) was purchased from Sigma–Aldrich (Shanghai, China). PHE stock solution was dissolved in acetone (4 mg mL^-1^). Colchicine, cytochalasin B, and sodium azide were purchased from Aladdin Reagent (Shanghai, China). Luria-Bertani (LB) medium: 10 g L^-1^ peptone, 5 g L^-1^ yeast extract and 10 g L^-1^ NaCl, pH 7.0. Potato-dextrose agar (PDA): 6 g L^-1^ potato extract powder, 20 g L^-1^ dextrose, 3 g L^-1^ KH_2_PO_4_, 1.5 g L^-1^ MgSO_4_, 8 mg L^-1^ VB_1_, 20 g L^-1^ agar, pH 5.6. Mineral salt medium (MSM): 0.5 g L^-1^ NaNO_3_, 1.0 g L^-1^ KH_2_PO_4_, 0.02 g L^-1^ CaCl_2_, 0.2 g L^-1^ MgSO_4_, 0.5 g L^-1^ (NH_4_)_2_SO_4_, 1.0 g L^-1^ NaH_2_PO_4_⋅H_2_O, pH 6.5. All the media were autoclaved at 121°C for 15 min. All the organic solvents used in the research were HPLC grade, and inorganic chemicals were analytical grade or higher.

### Inoculums Preparation

The isolation and collection of WF1 were based on the methods of Tian et al. (2008) and Zeng et al. (2010). Briefly, a 10-g PAHs-contaminated soil sample with 90 mL sterile water was shaken (150 rpm) for 2 h in a 250-mL conical flask, and then allowed it to settle for 30 min. The upper 1.0 mL of supernatant was transferred to a sterilized tube and diluted to 10^-5^ by gradient (1:10). Each 200-μL dilution of 10^-3^, 10^-4^, and 10^-5^ supernatant was spread on the solid MSM plates coated with a crystal layer of PHE. After incubation at 28°C for 7 days in dark, the developed colonies surrounded with clear zones were isolated as PHE-degrading bacteria. After repeatedly streaking in MSM plates with PHE, the single colony of WF1 was obtained. For inoculation, WF1 was incubated in LB medium for 12 h on a water orbital shaker (28 ± 0.5°C, 130 rpm). The cells were harvested by centrifugation (3K18, Sigma, Osterode, Germany) at 6010 × *g*, 4°C and for 5 min, and washed twice with sterilized MSM. The cell pellets were finally resuspended and adjusted with the sterilized MSM to an optical density (OD) at 600 nm of 1.0 before inoculating to the liquid cultures. The plate counting showed that the cell concentration of this bacterial suspension was ca. 1.5 × 10^8^ CFU mL^-1^.

The *P*. *chrysosporium* (collection number: 5.776) was purchased from China General Microbiological Culture Collection Center. After 7 days incubation on PDA plate at 28 ± 0.5°C, the spores were harvested and re-suspended with the sterilized MSM. The spore suspension (OD_600_, 1.0, ca. 8.2 × 10^6^ cells mL^-1^) was prepared for the inoculation. Additionally, 1.0 mL of spore suspension was incubated into 20 mL potato-dextrose medium (PDA without agar) for another 2 days to form mycelial pellets on a water orbital shaker (28 ± 0.5°C, 130 rpm). The mycelial pellets with size of about 0.2 cm were selected for subsequent inoculation. The dry weights (2.07 ± 0.13 mg) of three mycelial pellets with size of about 0.2 cm were almost equivalent to that of one mycelial pellet about 0.6 cm (2.22 ± 0.05 mg) which reported by Chen and Ding (2012) and Ding et al. (2013). The subsamples of WF1 and *P*. *chrysosporium* (spore and mycelial pellet) were inactivated by autoclaving under 121°C for 15 min. Both the active and inactivated microorganisms were used in the following biodegradation and biosorption experiments.

### Phenanthrene Biodegradation and Biosorption by Microorganisms

Because of its low aqueous solubility (ca. 1.3 mg L^-1^, 25°C), PHE concentrations in these experiments were kept about 1.0 mg L^-1^ in MSM to avoid the formation of solid-state PHE. The liquid culture with PHE (ca. 1.0 mg L^-1^) was prepared by adding PHE stock solution to sterilized MSM, followed by mixing and evaporation of acetone with magnetic stirrer in the aseptic environment. The prepared suspension (1.0 mL) of WF1, spores of *P*. *chrysosporium*, and three mycelial pellets of *P*. *chrysosporium* were inoculated into 9.0 mL liquid culture system containing PHE, respectively. To ensure the same background concentration of PHE, 1.0 mL sterilized MSM was added in the treatment of mycelial pellets. The 10.0 mL inoculated liquid culture system was placed in 50 mL brown glass tube with Teflon-lined silastic screw cap to avoid evaporation, and then incubated with rotation (130 rpm) at 28 ± 0.5°C in the dark with aeration for 1 min every 2 days. The same amounts of autoclaved microorganisms were added into the liquid culture systems containing PHE, which served as inactivated controls. Meanwhile, the uninoculated controls were treated by adding 1.0 mL sterilized MSM into 9.0 mL liquid culture system containing PHE. Samples were prepared in quintuplicate, that is, five replicates of each treatment were randomly selected for further analysis at each designated sampling point after incubation. There were 65 samples of WF1 treatment for 13 sampling points (0, 1, 2, 4, 6, 8, 10, 12, 24, 72, 168, 360, 720 h), and 40 samples of spores and mycelial pellets of *P. chrysosporium* for eight sampling points (0, 12, 24, 48, 72, 168, 360, 720 h), respectively.

The solution was separated from microorganisms by centrifugation (Multifuge 3s, Heraeus, Hanau, Germany) at 3990 × *g*, 4°C for 10 min, the supernatant was decanted as much as possible after centrifugation, and then added 2 mL MSM to wash the residual PHE on the surface of the cells with vortex and centrifugation. The supernatants were pooled and diluted with methanol (1:1, v:v), and the PHE residues in the solution were determined. The residual PHE was successively extracted from the microorganisms by ultrasonication for 30 min with 2 mL methanol. Specifically, the mycelial pellets of *P*. *chrysosporium* were whirled with micro glass beads for 5 min before ultrasonication to extensively break the cells. PHE residues in methanol were considered to be the biosorption amounts, such as PHE sorption on (adsorption) and in (absorption) the microorganisms (Thion et al., 2012).

Our previous experiments showed that PHE sorption on microorganisms (adsorption) were negligible, which was similar with the observation by Thion et al. (2012). After filtration through 0.22 μm PTFE filtration membranes, PHE residues in the diluted supernatants and methanol were determined separately by high performance liquid chromatography (HPLC). The biodegradation amounts were determined by mass difference in the uninoculated control and inoculated treatment (including PHE residues in the mixed supernatants and biosorption). The recoveries of PHE in the uninoculated controls were with a mean ± standard deviation value of 95.57 ± 3.52% during the incubation periods.

Simultaneously, the growth of WF1 was determined by testing the protein content with the method by Bradford (1976), and the growth of *P*. *chrysosporium* (spores and mycelial pellets) was monitored by the freeze-dried weight (Thion et al., 2012). In addition, the active microorganisms in the biodegradation and biosorption experiments, WF1 after incubated 2 h and the spores and mycelial pellets of *P*. *chrysosporium* after 2 days of incubation, were selected to visualize the auto-fluorescence of biosorbed PHE using two-photon excitation microscopy (TPEM) (LSM 710 NLO, Zeiss, Oberkochen, Germany) at excitation wavelength of 405 nm and emission wavelength of 410–481 nm.

### Inhibition Studies

In order to obtain the mechanisms involved in trans-membrane transport of PHE by microorganisms, similar methods mentioned in Section “Phenanthrene Biodegradation and Biosorption by Microorganisms” were applied to study the inhibition of PHE biodegradation and biosorption (two important processes after PHE transported into cells) by WF1 and *P*. *chrysosporium* (spores and mycelial pellets) using various blocking treatments including incubation temperature (4 ± 0.5°C), and the presence of sodium azide, colchicine, and cytochalasin B. As colchicine and cytochalasin B are known as the eukaryotic inhibitors of cytoskeleton modulating (Verdin et al., 2005), the bacteria WF1 was not treated by those two substances. The inoculums (mentioned in Section “Inoculums Preparation”) were pretreated with MSM containing sodium azide, colchicine, and cytochalasin B for 4 hrs, and then introduced into 50 mL brown glass tubes containing MSM and PHE (ca. 1.0 mg L^-1^). Powdered sodium azide was added directly to MSM at a final concentration of 100 mM (Fayeulle et al., 2014). Colchicine and cytochalasin B were added to MSM at a final concentration of 5 μM (Verdin et al., 2005). The samples were taken out at the designated time intervals of 0, 1, 2, 4, 6, 8, 10, 12, 24 h for WF1 and 0, 12, 24, 48, 72, 168, 360, 720 h for *P*. *chrysosporium* (spores and mycelial pellets) after incubation, and the following operation procedures were the same as mentioned in Section “Phenanthrene Biodegradation and Biosorption by Microorganisms”. The treatments without inhibitors were cultured at 28 ± 0.5°C and were used as the corresponding controls.

### PHE Analysis

Phenanthrene was analyzed by a Waters Alliance 2695–2475 HPLC system fitted with a Symmetry^®^ C18 column (5 μm, 3.9 mm × 150 mm) and a fluorescence detector (Waters, Milford, MA, USA). Mobile phase was methanol and water mixture (90:10, v:v) with a flow rate of 1 mL min^-1^, the column temperature was 30°C, and the injection volume was 50 μL. The excitation and emission wavelengths for determining PHE were 254 and 375 nm. The minimum detectable concentration for PHE in this study was 3.17 μg L^-1^, and the relative standard deviation (RSD) was 0.56% (*n* = 5).

### Statistical Analysis

First-order kinetic equation, *C* = *C*_0_ × e^-^*^kt^*, was applied to fit the degradation data. The time to reach 50% degradation (half-life time, *t*_1/2_) of PHE was further calculated from the formula *t*_1/2_ = ln2/k, where *C* is the residual PHE remaining in the solution at incubation time *t*, *C*_0_ is the initial concentration of PHE, and *k* is the first-order rate constant (Wang et al., 2010). Further, we assumed that the trans-membrane transport between inside and outside cells was occurred simultaneously at different rates. The concentrations of PHE inside and outside cells were used to calculate the intracellular and extracellular dissipation and transport of PHE by microorganisms based on the following equations by Kintecus V4.55 (Ianni, 2003):

$$\frac{dC_{in}}{dt}=\frac{S}{V_{in}}(K_{in}C_{out}-K_{out}C_{in})-K_{din}C_{in}$$

$$\frac{dC_{out}}{dt}=\frac{S}{V_{out}}(K_{out}C_{in}-K_{in}C_{out})-K_{dout}C_{out}$$

where *C*_in_ and *C*_out_ (mol/L) are the concentration of PHE inside and outside cells, *K*_in_ (m/s) is the transport rate of PHE from outside solution into cells, *K*_out_ (m/s) is the efflux rate of PHE from cells to outside solution, *S* (m^2^) is the exchange surface area, *V*_in_ (m^3^) is the volume of cell, *V*_out_ (m^3^) is the volume of outside solution, *K*_din_ and *K*_dout_ (s^-1^) are the intracellular and extracellular dissipation rate constants for PHE, and *t* (s) is the time. We employed a non-linear least-square regression algorithm to identify values of the kinetics parameters (*K*_in_*, K*_out_*, K*_din_, and *K*_dout_) that minimized the differences between modeled inside and outside concentrations of PHE and their corresponding measured values.

## Results

### Biodegradation of PHE by *Massilia* sp. WF1 and *P. chrysosporium*

**FIGURE 1:**
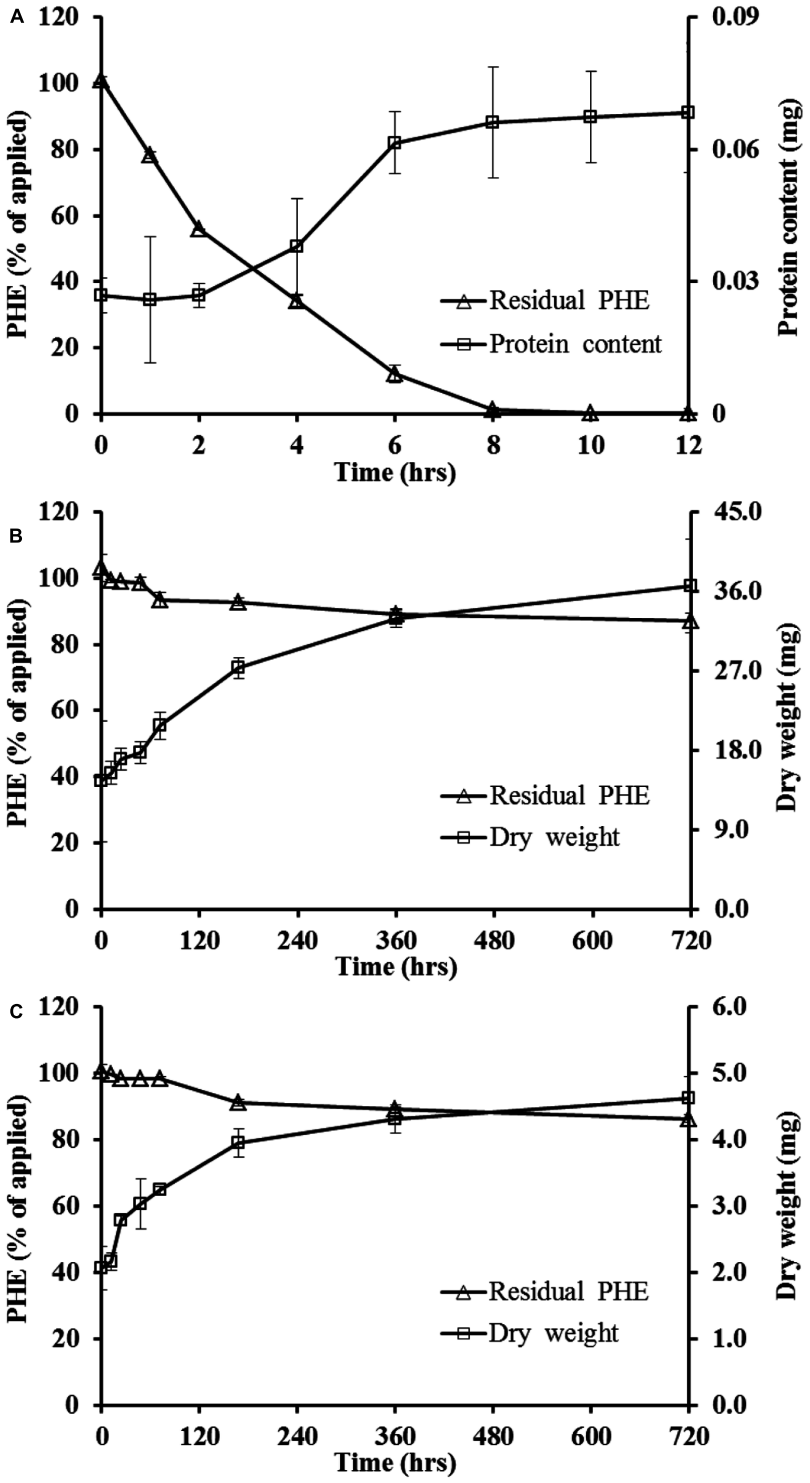
**Biodegradation dynamics of phenanthrene (PHE) by *Massilia* sp. WF1 **(A)**, spore **(B)**, and mycelial pellet **(C)** of *P. Chrysosporium* and the growth curve of microorganism in liquid solution cultures at 28°C.** Bars are ±standard deviation of three analyses.

It was shown in **Figure 1A**, WF1 utilized most of the PHE in the suspension as the sole carbon source after 12 h of incubation at 28°C. The PHE residual concentration decreased with incubation time and the data fitted well to the first-order kinetic equation (*R*^2^ = 0.970), with a *t*_1/2_ of about 2 h (**Table 1**). Corresponding to the decrease of remaining PHE with the incubation time, a slight increase of the protein content of WF1 was observed (**Figure 1A**). The discernible clearing zones appeared in the MSM agar plate with PHE (figure not shown) were also the evidence of PHE degradation by WF1. By comparison, PHE degraded rather slowly by active *P*. *chrysosporium*. During the 720 h incubation period, the biodegradation of PHE by *P*. *chrysosporium* gradually increased from 0.77 to 12.86% of the applied amount for the spores, and from 0.25 to 13.55% of the applied amount for the mycelial pellets with slight growth of both biomasses (**Figures 1B,C**). Both *t*_1/2_ values of PHE degraded by the spores and mycelial pellets were 86.64 days (**Table 1**).

**Table 1:** First-order kinetics equations and the corresponding half-life times of phenanthrene (PHE) biodegradation by *Massilia* sp. WF1, spore, and mycelial pellet of *Phanerochaete chrysosporium* at 28°C.

Microorganism	Exponential equation	*R*^2^	*t*_1/2_
*Massilia* sp. WF1	*C* = 1.086 e ^-0.342^*^t^*	0.970^∗∗^	2.03 h
Spore	*C* = 1.003 e^-0.008^*^t^*	0.989^∗∗^	86.64 d
Mycelial pellet	*C* = 0.999 e^-0.008^*^t^*	0.883^∗∗^	86.64 d


**C*: the residual PHE concentration at time *t*; *t*_1/2_: time to reach 50% degradation of PHE; ^∗∗^significant at 1% level.*

### Biosorption of PHE by *Massilia* sp. WF1 and *P. chrysosporium*

**FIGURE 2:**
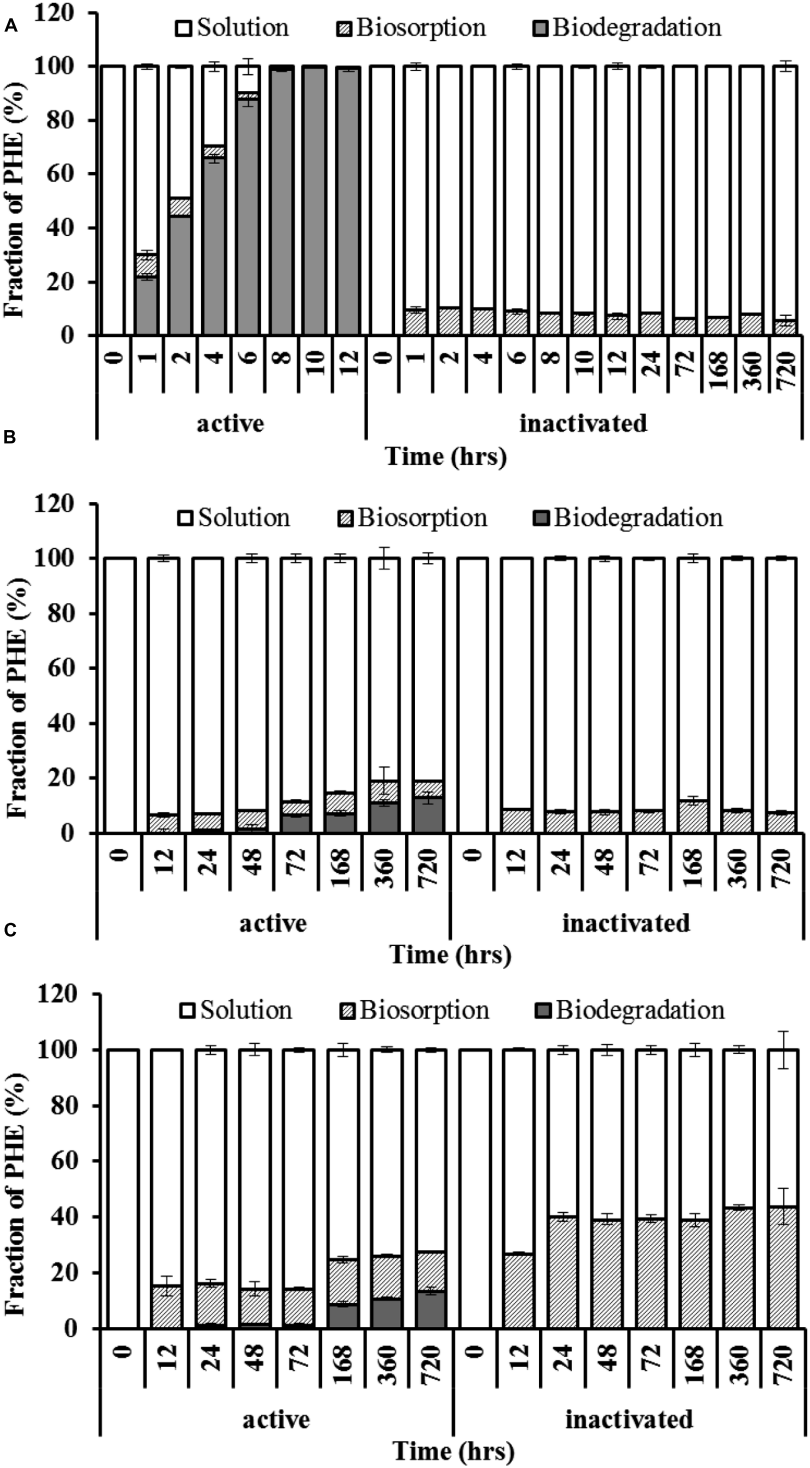
**Spatiotemporal distribution of PHE in liquid cultures with *Massilia* sp. WF1 **(A)**, spore **(B)**, and mycelial pellet **(C)** of *Phanerochaete chrysosporium* at 28°C.** Bars are ±standard deviation of five analyses.

**FIGURE 3:**
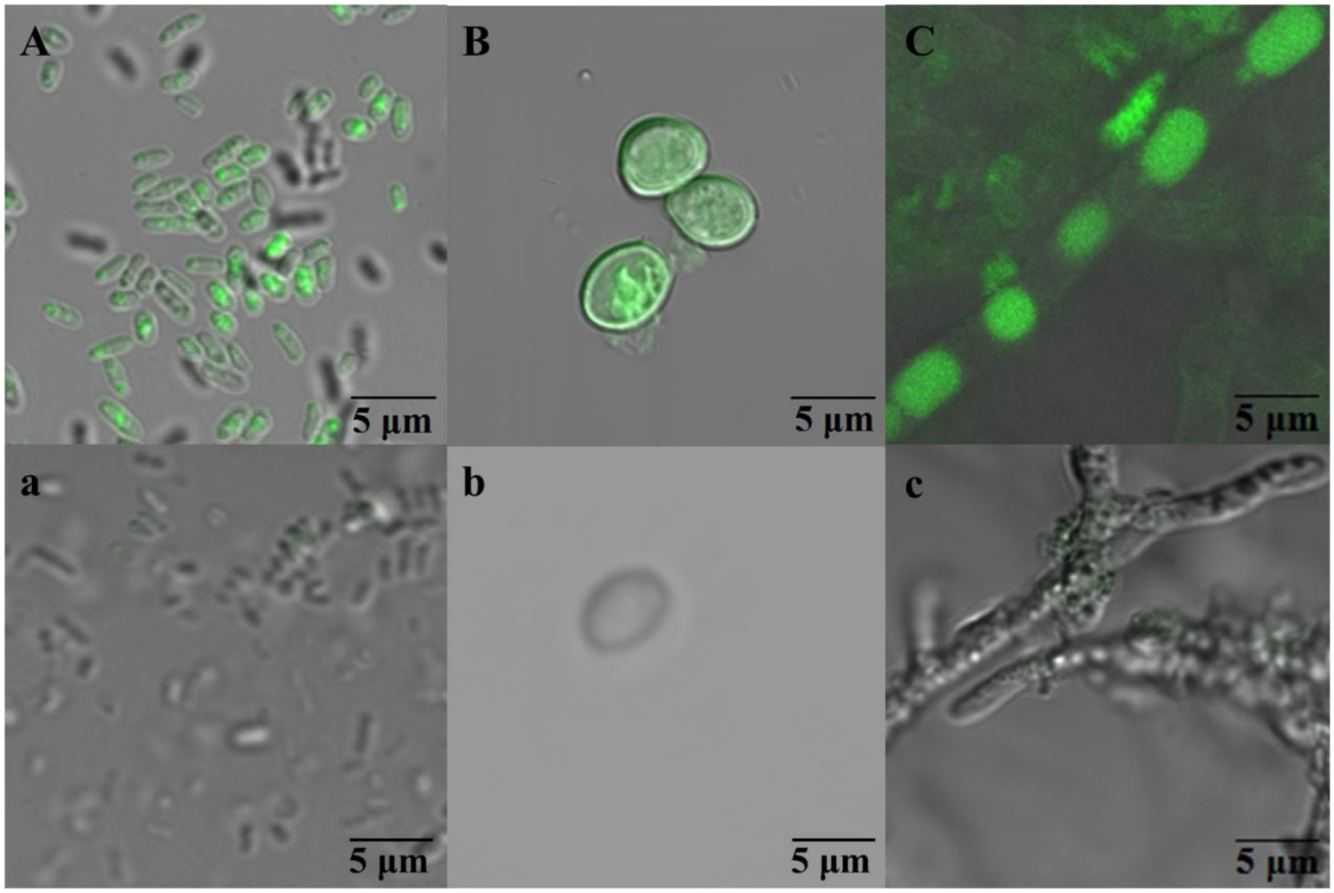
**The fluorescent signals observed in *Massilia* sp. WF1 **(A)**, spore **(B)**, and mycelial pellet **(C)** of *P. Chrysosporium* under two-photon excitation microscopy (TPEM).** WF1 **(A)**, the spores **(B)**, and mycelial pellets **(C)** of *Phanerochaete chrysosporium* were incubated 2 h (0.08 days), 3 days, and 3 days with PHE (ca. 1.0 mg L^-1^), respectively. WF1 **(a)**, the spores **(b)**, and mycelial pellets **(c)** of *P. chrysosporium* were incubated without PHE.

**Figure 2A** indicated that the PHE in solution reduced drastically and could not be detected at 8 h. However, the maximum biosorption of PHE in WF1 at 28°C was 8.03% of the applied amount at 1 h, then decreased with the incubation time, and biosorption was almost undetectable at 12 h. Biosorption of PHE by the active WF1 was further proved by the fluorescent signals observed in microorganisms under TPEM (**Figure 3A**). The biosorption amounts by the inactivated WF1 (about 7.30–10.02% of applied) were higher than that by the active ones and it did not change much during the incubation period (**Figure 2A**).

Compared with active WF1, the biosorption of PHE by the active *P*. *chrysosporium* at 28°C was relatively higher, especially in the mycelial pellets, and slighter change was observed during the incubation period (**Figures 2B,C**). As shown in **Figures 2B,C**, the residual PHE amounts in the solutions were higher than those of the biosorbed PHE and the PHE in solution decreased gradually with time. During the entire incubation period, the residual PHE amounts in the solutions for the active spores and mycelial pellets were 81.13–93.46% and 72.37–85.67% of applied, respectively, and the biosorbed PHE were 5.03–8.01% and 12.76–16.11% of the applied PHE, respectively (**Figures 2B,C**). The intracellular fluorescence of PHE was also clearly detected in the spores and mycelial pellets of *P*. *chrysosporium* by TPEM (**Figures 3B,C**). Additionally, the residual PHE in solutions for the inactivated spores and mycelial pellets were 88.19–92.67% and 56.29–73.09% of the applied PHE, respectively, and the biosorbed PHE reached 7.33–11.81% and 38.94–43.71% of the applied PHE, respectively (**Figures 2B,C**).

### Kinetics of PHE Dissipation and Transport by *Massilia* sp. WF1 and *P. chrysosporium*

Based on the measured values, we modeled the intracellular and extracellular PHE of the microorganisms to obtain the relevant kinetics parameters for PHE dissipation and transport (**Table 2**). The results clearly showed that both the intracellular (*K*_din_) and extracellular dissipation rate constants (*K*_dout_) of PHE for WF1 were higher than those of spores and mycelial pellets of *P*. *chrysosporium* at 28 and 4°C. There was very little difference between the dissipation of PHE by the active spores and mycelial pellets of *P*. *chrysosporium* at 28°C: the *K*_din_ of mycelial pellets was slightly larger than that of spores, but the *K*_dout_ of mycelial pellets was slightly smaller than that of spores (**Table 2**). However, the *K*_din_ was almost equal to the *K*_dout_ for WF1 at 28°C. Besides, the transport rate constant of PHE from outside solution into cells (*K_in_S/V_out_*) for WF1 was higher than the efflux rate constant of PHE from cells to outside solution (*K_out_S/V_in_*) at 28°C. In contrast, the *K_in_S/V_out_* values were smaller than the *K_out_S/V_in_* values, respectively, for the active spores and mycelial pellets of *P. chrysosporium* at both 28°C and 4°C (**Table 2**).

**Table 2:** Kinetics parameters for PHE dissipation and transport in the intracellular and extracellular of *Massilia* sp. WF1 and *P. chrysosporium* at 28 and 4°C.

Parameters	Temperature (°C)	*Massilia* sp. WF1	*P. chrysosporium*	
			
			Spore	Mycelial pellet
*K*_in_*S*/*V*_out_ (s^-1^)	28	0.34 × 10^-4^	0.24 × 10^-5^	0.67 × 10^-5^
	4	0.16 × 10^-4^	0.52 × 10^-5^	0.39 × 10^-5^
*K*_din_ (s^-1^)	28	0.14 × 10^-3^	0.34 × 10^-6^	0.43 × 10^-6^
	4	0.10 × 10^-5^	0.17 × 10^-6^	0.14 × 10^-6^
*K*_out_*S*/*V*_in_ (s^-1^)	28	0.31 × 10^-5^	0.32 × 10^-4^	0.38 × 10^-4^
	4	0.75 × 10^-4^	0.12 × 10^-4^	0.10 × 10^-4^
*K*_dout_ (s^-1^)	28	0.13 × 10^-3^	0.70 × 10^-7^	0.17 × 10^-7^
	4	0.16 × 10^-4^	0.18 × 10^-12^	0.30 × 10^-7^


*K_in_ (m/s): the transport rate of PHE from outside solution into cells, *K*_*out*_ (m/s): the efflux rate of PHE from cells to outside solution, *S* (m^2^): the exchange surface area, *V*_*in*_ (m^3^): the volume of cell, *V*_*out*_: the volume of outside solution, *K*_*din*_*S*/*V*_*out*_ (s^-1^): the transport rate constant of PHE from outside solution into cells, *K*_*dout*_*S*/*V*_*in*_ (s^-1^): the efflux rate constant of PHE from cells to outside solution, *K*_*din*_ and *K*_*dout*_ (s^-1^): the intracellular and extracellular dissipation rate constants for PHE, respectively.*

### The Trans-Membrane Transport Mechanisms of PHE by *Massilia* sp. WF1 and *P. chrysosporium*

**FIGURE 4:**
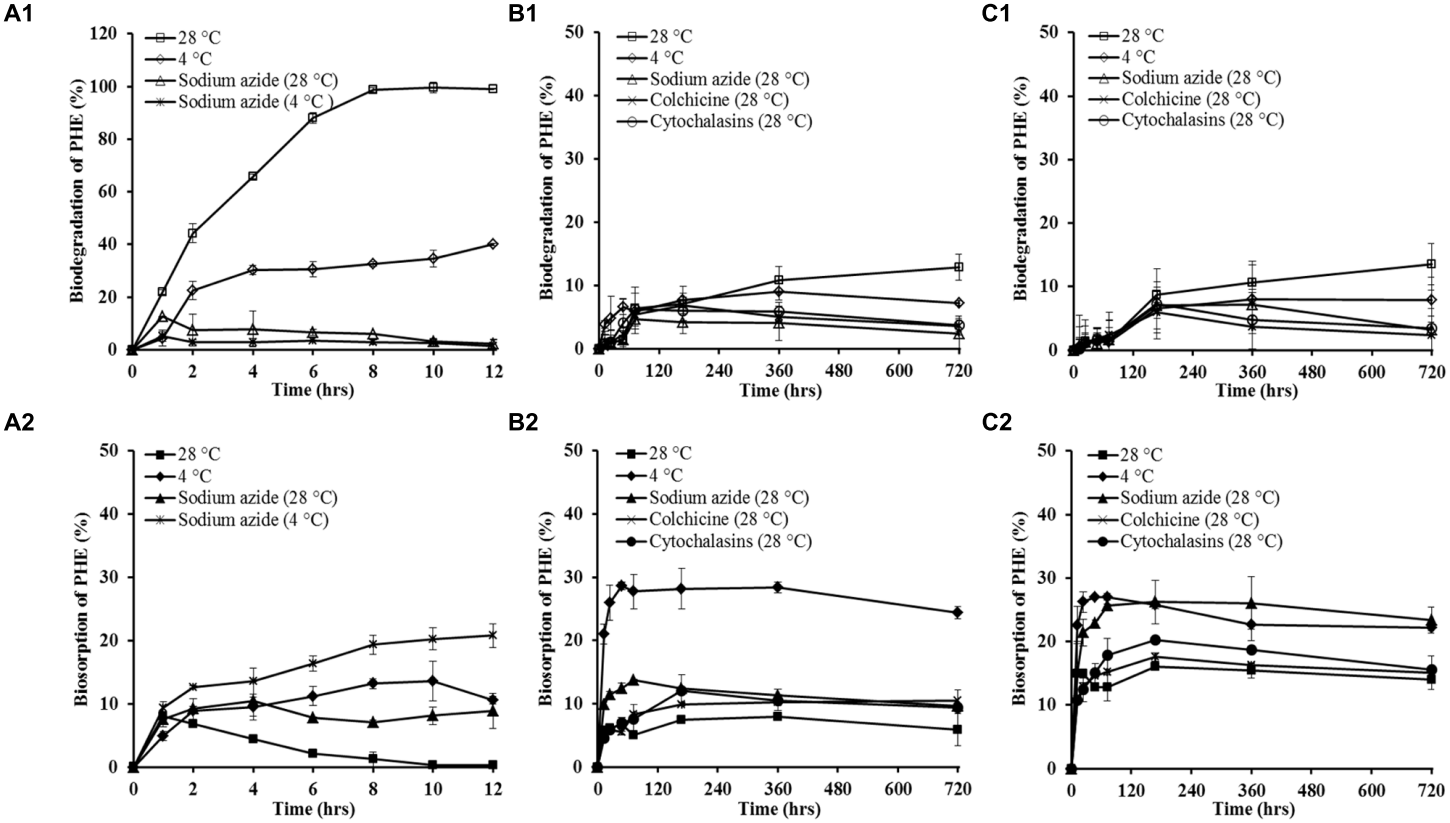
**The percentage of PHE biodegradation and biosorption by *Massilia* sp. WF1 **(A1,A2)**, spore **(B1,B2)**, and mycelial pellet **(C1,C2)** of *P. chrysosporium* under different inhibition treatments.** Bars are ±standard deviation of five analyses.

To learn the possible mechanisms involved in the trans-membrane transport of PHE by WF1 and *P*. *chrysosporium* (spore and mycelium pellet), the effects of some inhibition treatments on PHE biodegradation and biosorption were tested as the two processes are important after PHE transported into cells. As shown in **Figures 4A1,B1,C1**, the biodegradation of PHE by WF1 and *P*. *chrysosporium* (spore and mycelium pellet) under the different inhibition treatments were lower than that of the corresponding controls without inhibitors at 28°C, while the opposite trends were observed for the biosorption of PHE. Overall, the biosorbed PHE by *P*. *chrysosporium* was higher than that by WF1 under the different inhibition treatments. Except no significant difference for PHE biosorption between the spore and mycelium pellet at 4°C, the biosorbed PHE by mycelium pellet was higher than that by spore under the other inhibition treatments.

The *K*_din_ values of PHE for WF1, spores and mycelial pellets at 4°C were lower than those of at 28°C (**Table 2**). At 4°C, only 40.01, 8.97, and 8.01% of the applied PHE were degraded by WF1, spores, and mycelial pellets during the culture period, respectively (**Figures 4A1,B1,C1**), which were lower than that of the corresponding controls at 28°C (99.61, 12.86, and 13.55%). However, the biosorbed PHE in the microorganisms at 4°C were significantly higher (*P* < 0.01) than that of the other inhibition treatments and the corresponding controls (**Figures 4A2,B2,C2**). In the treatment of 4°C without sodium azide, the biosorbed PHE in WF1 increased gradually to 8.85% of the applied PHE at the first 2 h, and changed little from 2 to 12 h. While for *P*. *chrysosporium*, the biosorbed PHE increased rapidly at the beginning of the biosorption process (within 48 hrs), and then changed little from 48 to 720 h. As shown in **Table 2**, *K_in_S/V_out_* values changed little, but lower *K_out_S/V_in_* values for spore and mycelium pellet were observed at 4°C, compared with those of the corresponding controls without inhibitors at 28°C.

When treated with sodium azide, only 12.61 and 5.27% of the applied PHE were biodegraded by WF1 at 28 and 4°C, respectively (**Figure 4A1**). For the spores and mycelial pellets of *P*. *chrysosporium*, only 4.73 and 7.20% of the applied PHE were degraded at 28°C with sodium azide, respectively (**Figures 4B1,C1**). However, the biosorbed PHE by the microorganisms treated with sodium azide were significantly higher than those of the corresponding controls (*P* < 0.01). After treated with sodium azide, the biosorbed PHE by WF1 was enhanced to 8.95 at 28°C and 20.80% at 4°C within 12 h, respectively (**Figure 4A2**). For *P*. *chrysosporium*, the biosorbed PHE in spores and mycelial pellets were increased to 13.80 and 25.72% at the treatment of 28°C with sodium azide within 72 h, respectively (**Figures 4B2,C2**).

Similarly, the decreased biodegradation and increased biosorption of PHE by the spores and mycelial pellets of *P*. *chrysosporium* were observed under both the treatments of colchicine and cytochalasin B (**Figures 4B1,C1,B2,C2**).

## Discussion

### Biodegradation of PHE by *Massilia* sp. WF1 and *P. chrysosporium*

This study clearly showed that the strain WF1 used PHE as the sole carbon and energy source, and it degraded PHE completely in a short time period (**Figures 1A** and **2A**). The almost equivalent values of intracellular (*K*_din_) and extracellular dissipation rate constants (*K*_dout_) of PHE for WF1 indicated that the fast PHE transportation from outside solution into cells and the rapid degradation in cells of WF1. Moreover, our preliminary results further indicated that WF1 could degrade about 97 and 60% of PHE, respectively, in 2 days in 10 mL of liquid culture systems with 200, 400 mg L^-1^ of PHE as the sole carbon source (data not shown). The earlier reported biodegradation percentages of PHE in 7–30 days for different microorganisms were 22–99.8% in liquid cultures containing of 10–500 mg L^-1^ PHE (Romero et al., 1998; Moody et al., 2001; Vacca et al., 2005; Thion et al., 2012). Thus it can be concluded that WF1 has high degradation ability and tolerance to high concentration of PHE. It is critical to further investigate what pathways, mechanisms and enzymes involve in high efficient PHE biodegradation by *Massilia* sp. WF1 in the nearest future.

By contrast, the experimental data showed that the active spores and mycelial pellets of *P*. *Chrysosporium* had low biodegradation ability of PHE (only about 13% in 720 h) in the liquid culture with ca. 1.0 mg L^-1^ of PHE as the sole carbon source (**Figures 1B,C** and **2B,C**). However, several previous studies demonstrated high levels of PHE degradation by *P*. *Chrysosporium* under ligninolytic, nutrient-sufficient, or other induced culture conditions by means of the ligninolytic exocellular enzymes or intracellular catabolism processes (Sutherland et al., 1991; van den Brink et al., 1998; Syed and Yadav, 2012). The reported PHE biodegradation percentages of *P. Chrysosporium* were 6.7–84.77% in 1–60 days in liquid cultures with 1.0–20 mg L^-1^ PHE when nutrients were present (van den Brink et al., 1998; Zhang et al., 2008; Ning et al., 2010). Accordingly, we attributed the low PHE biodegradation ability of *P*. *Chrysosporium* in this study to the deficiency of nutrients and/ or substrates in liquid cultures. As a sole carbon source, the low concentration of PHE (ca. 1.0 mg L^-1^) was not enough to induce many relevant degrading enzymes secreted by *P*. *Chrysosporium*. Thus, it is better to add other carbon sources or nutrients to improve PAHs biodegradation efficiency of *P*. *Chrysosporium*.

### Biosorption of PHE by *Massilia* sp. WF1 and *P. chrysosporium*

After entering into cells, the PHE can be biosorbed by the binding sites (Fayeulle et al., 2014). The biosorbed PHE by active WF1 were relatively low and nearly used up at 12 h (**Figure 2A**). It suggested that the biosorbed PHE in WF1 can be readily accessible for biodegradation with prolonging the incubation time. This agreed with the report of Chen et al. (2010), they found that the active microorganisms can be viewed both as a bio-sorbent that retains OPs as well as a bioreactor that degrades the OPs.

However, the biosorbed PHE in *P*. *Chrysosporium* was relatively high, especially in mycelial pellets. It can be attributed to the special physiology characteristics of this strain. The abundant conjugated structures (C=C and aromatic components), numerous chemical groups (–OH, –COO–, O–C=O, –NH_2_, CO–NH) and high carbon content in *P*. *Chrysosporium* may act as active adsorption sites and adsorb planar structure PHE by the π–π and electron donor–acceptor interactions (Chen et al., 2010; Gu et al., 2015). Besides, the lipid vesicles in hyphae were also the sites to accumulate PHE (Verdin et al., 2005; Furuno et al., 2012; Thion et al., 2012). As shown in **Figures 2B,C**, the biosorbed PHE in *P*. *Chrysosporium* changed slightly during the incubation period, which may contribute to the low biodegradation of PHE by *P*. *Chrysosporium* when PHE provided as the sole carbon source. According to previous literatures (Romero et al., 1998; Moody et al., 2001; Bago et al., 2002; Verdin et al., 2005; Wu et al., 2009; Chen et al., 2010; Furuno et al., 2012; Thion et al., 2012), the biosorbed PHE would finally be degraded with prolonging the incubation time and/ or added with other carbon sources.

Moreover, the difference of biosorbed PHE between the inactivated and active microorganisms further showed the existence of intracellular degradation of PHE by the microorganisms (**Figures 2A–C**). For the enhanced biosorbed PHE in the inactivated microorganisms, it might be partly attributed to the loss of biodegradation capacity after autoclaving (Chen et al., 2010). Besides, the increase of surface area and porosity of the disrupted cells were also important reasons for higher PHE biosorption of the inactivated microorganisms (Aksu, 2005).

### Kinetics of PHE Dissipation and Transport by *Massilia* sp. WF1 and *P. chrysosporium*

In the liquid culture with ca. 1.0 mg L^-1^ of PHE as the sole carbon source, the extracellular PHE can be transported from outside solution into cells and also can be degraded by the secreted extracellular enzymes. To the best of our knowledge, the ligninolytic fungi have been characterized by the production of some extracellular enzymes related to OPs degradation (Bamforth and Singleton, 2005; Haritash and Kaushik, 2009; Zhang et al., 2010; Thion et al., 2012; Hong et al., 2013) but there were hardly any reports on extracellular biodegradation of PAHs or other OPs by bacteria (Xu et al., 2013). Our previous experiments have indicated that neither WF1 nor *P*. *chrysosporium* has the extracellular biodegradation capacity (data not shown). Thus, the extracellular PHE dissipation mainly attributed to the trans-membrane transport from solution into cells. After entering into cells, the PHE can be decreased by the intracellular biodegradation and transportation from cells to outside solution (efflux) as PAHs were toxic compounds to many microorganisms (Bugg et al., 2000; Li et al., 2014).

For WF1, the transport rate constant of PHE from outside solution into cells (*K*_in_*S/V*_out_) was higher than the efflux rate constant of PHE from cells to outside solution (*K*_out_*S/V*_in_, **Table 2**). The almost equivalent values of intracellular (*K*_din_) and extracellular dissipation rate constants (*K*_dout_) of PHE for WF1 were higher than those of the spores and mycelial pellets of *P*. *chrysosporium* (**Table 2**). These phenomena combined with the high biodegradation capacity of PHE by WF1 indicated that the amount of PHE that transported into cells decreased mainly due to biodegradation, as reported by earlier investigation (Chen et al., 2010; Fayeulle et al., 2014; Li et al., 2014).

However, for the spores and mycelial pellets of *P*. *Chrysosporium*, the transport rate constants of PHE from outside solution into cells (*K*_in_*S/V*_out_) were lower than those of efflux rate constant (*K*_out_*S/V*_in_, it showed that the efflux of PHE was more easily occurred compared with the transport from outside solution into cells. In addition, the *K*_din_ values were higher than the *K*_dout_ values for the spores and mycelial pellets (**Table 2**), these results combined with the low PHE biodegradation capacity (**Figures 2B,C**) further proved the slow transformation rate of PHE, and the concentration of PHE which transported into cells can be decreased mainly by the active efflux (Bugg et al., 2000; Huang et al., 2014; Li et al., 2014).

### The Trans-Membrane Transport Mechanisms of PHE by *Massilia* sp. WF1 and *P. chrysosporium*

The mechanism for the trans-membrane transport of hydrophobic organic compounds (HOCs) into microorganism is far from clear and actually quite controversial. The three main hypotheses for hydrocarbons transport into cells include the active transport mechanism (Fayeulle et al., 2014), the passive transport mechanism (Verdin et al., 2005), and the endocytotic internalization-like mechanism (Luo et al., 2013; Fayeulle et al., 2014). Fayeulle et al. (2014) indicated that PHE biosorption was a result of the trans-membrane transport of PHE by one or more of the three mechanisms. Besides, the PHE transported from outside solution into cells can also be degraded intracellularly and transported to outside of cells as PAHs were toxic to many microorganisms (Bugg et al., 2000; Cheng et al., 2014; Huang et al., 2014; Li et al., 2014).

At 4°C, the low metabolic activity, poor membrane fluidity and slow molecular motion of the cells were observed (Luo et al., 2013; Dedaldechamp et al., 2014). Thus, decrease in *K*_din_ values (**Table 2**) and lower biodegradation of PHE by WF1 and *P*. *Chrysosporium* (spores and mycelial pellets) were observed at 4°C during the culture period compared with those of the corresponding controls without inhibitors at 28°C (**Figures 4A1,B1,C1**). It has reported that the effect of active trans-membrane transport was greatly weakened and the endocytosis or vesicular transport was almost inhibited at 4°C (Verdin et al., 2005; Luo et al., 2013). However, in the present study, the incubation temperature of 4°C did not inhibit PHE trans-membrane transport into cells. The *K*_in_*S/V*_out_ values of PHE for both microorganisms did not change much at 4°C compared with those of the corresponding controls without inhibitors at 28°C (**Table 2**). Moreover, the high PHE biosorption by both microorganisms were also observed (**Figures 4A2,B2,C2**). It suggested that the trans-membrane transport of PHE by the microorganisms was energy-independent and the possibility of endocytosis or vesicular transport can be excluded. Similar phenomena were reported by Verdin et al. (2005) who found that the passive trans-membrane transport mechanisms were involved in the processes of PHE entering into the microorganisms.

In addition to the decreased biodegradation, the higher PHE biosorption at 4°C can also be explained by the influence of temperature on PHE biosorption as the exothermic characteristic of PHE biosorption by *P*. *Chrysosporium* (Gu et al., 2015). Besides, the weakened active efflux (*K*_out_*S/V*_in_, **Table 2**) for the spores and mycelial pellets of *P*. *Chrysosporium* at 4°C may also be one of the main reasons for the higher PHE biosorption, as reported by Bugg et al. (2000). Thus, it is reasonable to speculate that the decrease of temperature can lead to the increase of PHE sorption capability by the microorganisms. With regard to the lower *K*_out_*S/V*_in_ value of PHE for WF1 at 28°C compared with that at 4°C (**Table 2**), it is attributed to the high PHE biodegradation efficiency of WF1 at 28°C, and the active efflux was not in full functioning at this temperature.

Sodium azide is a metabolic inhibitor that blocks cellular ATP synthesis (Verdin et al., 2005; Luo et al., 2013; Fayeulle et al., 2014; Huang et al., 2014). When the microorganisms were treated with sodium azide, the metabolic activities including active trans-membrane transport can be inhibited, resulting in the lower PHE biodegradation of WF1 and *P*. *Chrysosporium* (**Figures 4A1,B1,C1**). After treated with sodium azide, the biosorbed PHE (**Figures 4A2,B2,C2**) in the microorganisms were even higher (*P* < 0.01) than those in the corresponding controls. Thus, the treatment of sodium azide did not prevent the process of PHE trans-membrane transport into cells. It also suggested that the process of PHE entering into the cells was not an energy-dependent mechanism, but a passive one (Verdin et al., 2005). Moreover, earlier reports indicated that the active efflux of PHE might also be weakened for the microorganisms treated with sodium azide (Bugg et al., 2000; Huang et al., 2014; Li et al., 2014). Therefore, more PHE were accumulated in WF1 and *P*. *Chrysosporium* (**Figures 4A2,B2,C2**).

Besides, colchicine and cytochalasin B are known as the eukaryotic inhibitors of cytoskeleton modulating: microtubules and actin filaments, respectively (Verdin et al., 2005; Luo et al., 2013; Fayeulle et al., 2014). Some researchers reported that cytoskeleton can be involved in the synthesis and motion of lipid vesicles (Verdin et al., 2005; Fayeulle et al., 2014). Thus, the addition of colchicine and cytochalasin B to the culture medium of *P*. *Chrysosporium* (spores and mycelial pellets) can inhibit the vesicular transport of PHE to outside of cells. The higher PHE biosorption by *P*. *Chrysosporium* under these inhibition treatments further indicated the inhibited active efflux and passive transport of PHE by *P*. *Chrysosporium*. The higher toxicity of the inhibitors to mycelial pellets than that of spores due to their special structures (Dufrene et al., 1999), which might contribute to the lower active efflux and higher biosorbed PHE in mycelial pellets than those in spores.

## Conclusion

This research shows that the biodegradation of PHE by WF1 was significantly greater than that by *P. chrysosporium*, whereas *P. chrysosporium* can biosorb more PHE than WF1. In addition, the amount of PHE that transported from outside solution into cells of WF1 and *P*. *Chrysosporium* decreased mainly due to rapid biodegradation and active efflux of the microorganisms, respectively. Besides, the inhibition treatments did not prevent PHE entering into WF1 and *P*. *Chrysosporium*, thus, the passive trans-membrane transport mechanism was involved in PHE entering into the cells of WF1 and *P*. *Chrysosporium*. Considering the higher abilities of PHE biodegradation by WF1 and PHE biosorption by *P*. *Chrysosporium*, it is of great value to develop a co-culture system with the complementary advantages of WF1 and *P*. *Chrysosporium* for improving PAHs bioremediation in environment.

## Author Contributions

HG planned and designed the study, participated in the sampling and the running of experiments, wrote and revised the manuscript. JL participated in the sampling and the running of experiments. HW planned and designed the study, obtained funding, reviewed the manuscript and final approved of the version to be published. YY participated in the some data analyses. LW reviewed the manuscript and gave some suggestions. JW reviewed the manuscript. JX provided instruments platform and reviewed the manuscript.

## Conflict of Interest Statement

The authors declare that the research was conducted in the absence of any commercial or financial relationships that could be construed as a potential conflict of interest.
